# Supplementary material for: Dynalign II: common secondary structure prediction for RNA homologs with domain insertions
Source: Nucleic Acids Res. 2014 Nov 21;42(22):13939–48. doi: 10.1093/nar/gku1172 (PMC4267632; doi:10.1093/nar/gku1172)
Supplement: SUPPLEMENTARY DATA [file supp_gku1172_nar-02021-z-2014-File012.zip › manual/Overview/Contributors.html]

RNAstructure Installation and Overview: Running the JAVA GUI Interface


|  |  |  |
| --- | --- | --- |
|  | RNAstructure Installation and Overview Contributors | - Contents - Index |
| David Mathews wrote many of the original components, the Windows GUI, and the first components of the class library.  Stas Bellaousov contributed to ProbKnot.  Stas Bellaousov, Wayne Huggins, and Christine Hajdin wrote ShapeKnots.  Rhiju Das and Pablo Cordero contributed the ability of efn2 to use SHAPE data and the ability to apply pairing energy restraints in Fold. Additionally, they contributed a knowledge-based method for determining stacking restraints from chemical mapping data.  Laura DiChiacchio wrote the DuplexFold algorithm.  Yinghan Fu wrote Multifind and revised Dynalign to make Dynalign II.  Jason Gloor wrote the first implementation of MaxExpect.  Arif Harmanci wrote PARTS, TurboFold, and the pairwise alignment HMM.  Jim Hart wrote NAPSS.  Joshua Keegan wrote the first configuration file implementation, used by Dynalign, PARTS, TurboFold, and Multilign.  John Lu extended OligoWalk, wrote the OligoWalk command line interface, added the O(N^3) code for predicting internal loops of any size in free energy minimization and the partition function, and wrote the OligoWalk web server.  Jacob Mainzer wrote the extended\_double type and added single-stranded SHAPE constraints to free energy minimization.  Michael Mierzejewski wrote many of the SMP versions of the programs, with contributions by David Mathews.  Jessica Reuter wrote the JAVA GUI, the Makefile system, CircleCompare and most of the command line interfaces.  Harry Stern wrote partition-cuda.  Paul Tymann, Glenn Katzen, Chris Connett, and Andrew Yohn, Computer Science, Rochester Institute of Technology, contributed changes that accelerated Dynalign and also wrote the SMP version of Dynalign.  Kevin Weeks and Katie Deigan contributed to the methods for using SHAPE data to restrain secondary structure prediction.  Kevin Weeks and Christine Hajdin contributed to the design of CircleCompare.  Kevin Weeks, Christine Hajdin, and Stas Bellaousov determined the energy function and best parameters used in ShapeKnots.  Zhenjiang Xu wrote the Multilign algorithm. | | |
| Visit The Mathews Lab RNAstructure Page for updates and latest information. | | |
